# Nutritional Strategies for the Management of Type 2 Diabetes Mellitus: A Narrative Review

**DOI:** 10.3390/nu15245096

**Published:** 2023-12-13

**Authors:** Tatiana Palotta Minari, Lúcia Helena Bonalume Tácito, Louise Buonalumi Tácito Yugar, Sílvia Elaine Ferreira-Melo, Carolina Freitas Manzano, Antônio Carlos Pires, Heitor Moreno, José Fernando Vilela-Martin, Luciana Neves Cosenso-Martin, Juan Carlos Yugar-Toledo

**Affiliations:** 1 Department of Hypertension, State Faculty of Medicine of São José do Rio Preto (FAMERP), São José do Rio Preto 15090-000, SP, Brazil; 2 Department of Endocrinology, State Faculty of Medicine of São José do Rio Preto (FAMERP), São José do Rio Preto 15090-000, SP, Brazil; 3 School of Medical Sciences, State University of Campinas (UNICAMP), Campinas 13083-887, SP, Brazil; 4 Cardiovascular Pharmacology & Hypertension Laboratory, School of Medical Sciences, State University of Campinas (UNICAMP), Campinas 13083-887, SP, Brazil

**Keywords:** nutritional interventions, nutritional therapy, type 2 diabetes mellitus, nutritional strategies, dietary prescription

## Abstract

Background: Thinking about greater adherence to dietary planning, it is extremely important to be aware of all nutritional strategies and dietary prescriptions available in the literature, and of which of them is the most efficient for the management of T2DM. Methods: A search was carried out in 2023 for randomized clinical trials, systematic reviews, meta-analyses, and guidelines in the following databases: Pubmed, Scielo, Web of Science, CrossRef and Google Scholar. In total, 202 articles were collected and analyzed. The period of publications was 1983–2023. Results: There is still no consensus on what the best nutritional strategy or ideal dietary prescription is, and individuality is necessary. In any case, these references suggest that Mediterranean Diet may of greater interest for the management of T2DM, with the following recommended dietary prescription: 40–50% carbohydrates; 15–25% proteins; 25–35% fats (<7% saturated, 10% polyunsaturated, and 10% monounsaturated); at least 14 g of fiber for every 1000 kcal consumed; and <2300 mg sodium. Conclusions: Individuality is the gold standard for dietary prescriptions, however, the Mediterranean diet with low levels of carbohydrates and fats seems to be the most promising strategy for the management of T2DM.

## 1. Introduction

Type 2 diabetes mellitus (T2DM) is a highly prevalent chronic disease worldwide, and represents one of the biggest public health problems of the 21st century. Its high incidence and prevalence are attributed to population aging and lifestyle, which is characterized by physical inactivity and eating habits that predispose individuals to obesity and metabolic syndrome [1].

At the same time, this epidemiological scenario is worsening with the increase in obesity rates [2]. According to the literature, obesity can predispose individuals to the development of chronic noncommunicable diseases such as T2DM, hypertension and cardiovascular diseases that probably reflect changes in lifestyle characterized by an increase in energy intake and a reduction in physical exercise [2,3,4].

For patients with T2DM and who are overweight, progressive weight loss is recommended to improve quality of life and treatment. This recommendation is based on short-term studies that point to the several benefits of the weight loss process, including improvements in glycemic modulation, cardiorespiratory markers, and quality of life [3,5,6,7]. However, the big question is “what is the patient’s motivation to form new healthy habits and control the disease in the long term?” [8].

Thinking about greater adherence to dietary planning, it is important for the nutritionist to be aware of all dietary prescriptions and nutritional strategies available in the literature. Another step would be a critical analysis of them, thus verifying which would be the most efficient in the management of T2DM.

## 2. Methods

A search was carried out in 2023 for randomized clinical trials, systematic reviews, meta-analyses, and guidelines in the following databases: Pubmed, Scielo, Web of Science, CrossRef, and Google Scholar. In total, 202 articles were collected and analyzed. The period of publications was 1983–2023. The MeSH indexed terms searched were nutritional interventions, nutritional therapy, type 2 diabetes mellitus, nutritional strategies and dietary prescription.

## 3. Results

### 3.1. The Importance of Individuality in Nutritional Management of T2DM

The treatment of patients with T2DM can be controlled through structured lifestyle programs, which include dietary education, intensive interdisciplinary therapy and physical exercise [6,9,10,11,12]. The individuality of the treatment and the synergy of interdisciplinary team (nutritionist, endocrinologist, cardiologist, physical educator, and psychologist) are fundamental to increase the patient’s quality of life. Another relevant factor is maintaining empathy and trust between professionals and patients, including the person with T2DM, who is the protagonist of the intervention [9,11,12,13,14,15,16].

The strongest evidence for T2DM prevention includes intensive lifestyle intervention resulting in weight loss, reduced incidence of T2DM in overweight adults, and decreased glucose tolerance over three years [17]. Other studies with lifestyle interventions have shown a reduction of 43% in T2DM progression over 20 years [18,19], of 34% over 10 years [17], and of 27% over 15 years [20], and have demonstrated a reduction in all-cause cardiovascular mortality [21].

Over the years, nutritional interventions have gained increasing prominence in the prevention, treatment, and maintenance of the disease. Strong evidence supports the high effectiveness and cost-effectiveness of nutritional therapy for the treatment of T2DM. Therefore, it is extremely important that all members of the healthcare team know the benefits of improving the quality of life of T2DM patients in the long term [6,22,23,24,25,26].

Nutritional management must consider life cycles, nutritional diagnosis, eating habits, individual characteristics, sociocultural issues, the patient’s economic situation, metabolic profile, use of drugs, physical exercise, and other factors. The dietary prescription, nutritional strategy, and eating plan must be individualized, taking into account the following objectives: to improve glycemic markers, to increase weight loss, and to reduce cardiovascular risk [9,27,28,29,30,31].

A generic healthy living plan is not enough to prevent and control T2DM, being unrealistic and incompatible with the peculiarities of each patient’s clinical case. This review provides clarity about the many dietary choices and patterns that can help people achieve health and a better quality of life [9,27,28,29,30,31]. Individualized nutritional management with a multidisciplinary approach can help reduce glycated hemoglobin (HbA1C), presenting similar or even greater results than would be expected with pharmacological treatment for T2DM. According to evidence, reductions in HbA1C could reach up to 2.0% in patients with T2DM in 3–6 months [32,33]. The cost–effect relationship of various lifestyle interventions for diabetes prevention and control have also been documented in several other studies [32,33,34,35,36].

### 3.2. Dietary Prescription and Macronutrient Distribution

Evidence suggests that there is no nutritional strategy or ideal percentage of calories from carbohydrates, proteins, and fats for all people with T2DM. Therefore, the type of strategy and distribution of macronutrients should be based on an individualized assessment of current eating patterns, preferences, and metabolic goals. The strategy should include an individualized dietary prescription on carbohydrate intake and glycemic self-monitoring to improve the patient’s meal timing, portioning, and food choices. Health professionals must advise the correct use of medications and regular physical exercise. Moreover, patients with T2DM should also be encouraged to consume fiber, vegetables, legumes (beans, peas, and lentils), fruits, and whole grains for the reduction of HbA1C [9,27,28,36,37,38].

#### 3.2.1. Carbohydrates

Carbohydrate is a source of energy used by the body, and is responsible for the postprandial increase in blood glucose [36,37,38,39]. Foods that contain carbohydrates (sugars, starches, or fiber) have a wide range of effects on the individual glycemic response. Some types of carbohydrate prolong the increase and slow the decrease in blood glucose concentrations, while others cause a rapid rise followed by a rapid fall [40].

Food sources of carbohydrates, especially those made up predominantly of dietary fiber, vitamins, and minerals (those low in added sugars, fats, and sodium) should be highlighted in individualized eating plans [32,36,37,38,41]. The amount of carbohydrates needed for optimal health in patients with T2DM is still uncertain, and studies vary greatly in the types of approaches and percentages of prescriptions. However, the literature indicates that dietary intake of carbohydrates in T2DM should be around 130 g/day or 40–50% of carbohydrates within the total energy value (TEV), aiming mainly to meet brain glucose requirements. The body’s other metabolic processes could be supplied by other energy substrates, such as fatty acids, amino acids (glycogenolysis and gluconeogenesis), and ketone bodies (ketogenesis) [39,40,41,42].

-Glycemic Index and Glycemic Load

The use of glycemic index (GI) and glycemic load (GL) to classify foods rich in carbohydrates according to their effects on glycemia still remains of interest to the scientific community, especially in the management of T2DM. According to studies, GI and GL could provide a prediction of postprandial blood glucose, the glycemic response curve, and may also classify carbohydrate-rich foods according to their postprandial glycemic response [43].

However, two systematic reviews with GI reported no significant impact on HbA1C, and had mixed results on fasting glucose [32,40]. Furthermore, studies use various definitions to classify low- and high-GI foods, leading to uncertainty regarding their usefulness in clinical care, and this would be a too simplistic metric for consideration in the management of T2DM. The authors discuss that several factors can have an impact, such as the following: (1) Individual glycemic response; (2) The preparation method and cooking time of foods that are sources of carbohydrates; (3) The addition of other food types to the meal, such as vegetables, legumes, proteins, and poly and monounsaturated fats; (4) The addition of drinks to the meal; (5) The order of food intake; (6) The gut microbiota [44,45,46,47,48].

A recent study showed that the order of food intake during a meal affects postprandial glucose and insulin peaks in pre-diabetes. The researchers suggest that eating vegetables and protein-rich foods at the beginning of meals and subsequently eating carbohydrate-rich foods may present an innovative and economically accessible behavioral strategy to reduce postprandial glycemic and insulinemic peaks in pre-diabetes, or to prevent T2DM [46].

Another study monitored glucose levels in 800 participants, and their glycemic responses were measured in 46.898 meals. A high individual variability was found in the different responses to the identical meals offered. Some participants had greater postprandial blood sugar spikes after eating cookies, and others did not have such good responses. Other participants did not show an increase in postprandial blood glucose after eating bananas, and others showed an increase. These findings suggest that universal dietary recommendations may have limited usefulness, because there are several factors that can alter these glycemic responses, such as genetics, sex, age, diet, mental health, and even composition of the gut microbiota [45]. Therefore, individuality must be taken into consideration when creating a dietary prescription, especially for T2DM patients who continuously monitor their blood glucose levels [45,46].

According to guidelines, glycemic load has more relevance in treatment of T2DM, as it takes into account the amount of carbohydrates presents within a 100 g portion [9,49]. Counting carbohydrates in the distribution of the patient’s meals is essential when putting together dietary planning. The ‘Carbohydrate Counting Manual’ is a great guide formulated by the Brazilian Diabetes Society that provides a resource for patients with T1DM or T2DM to educate themselves and understand the disease [49]. Therefore, carbohydrate counting, that is, knowing the amount of carbohydrates present in a 100 g portion (glycemic load), seems to be more important for dietary management than analyzing the GI alone. In this way, foods that are sources of carbohydrates (fruits, tubers, cereals, and whole grains) can be included in the dietary plan, as long as they are within the daily carbohydrate quantification and are combined with other foods [9,47,49].

#### 3.2.2. Proteins

The references show a wide disparity in the percentage of protein prescription in the management of T2DM. Some comparisons of protein amounts did not demonstrate differences in results related to T2DM. Prescriptions ranged from 15 to 20% of TEV, but more studies are needed [50,51,52,53,54]. It is worth remembering that in individuals with T1DM and T2DM, the intake of some foods that are sources of protein, such as dairy products, can also increase the postprandial insulin response. Therefore, the use of high-carbohydrate hypercaloric diets in conjunction with high protein should be avoided when treating patients, due to the potential increase insulin [9].

However, high-protein diets are advocated by some researchers as a strategy to facilitate weight loss, compared to other energy restriction strategies. The hypothesis would be that patients with T2DM could benefit from high-protein diets due to better blood glucose control. One study compared 30% protein intake within TEV vs. 15% protein within TEV for 12 weeks. The results showed a reduction in weight, fasting glucose, and medication use in the group that consumed 30% of their TEV in protein [54]. Other studies lasting 4 to 24 weeks reported that high-protein diets (25–32% of TEV) resulted in 2 kg more weight loss and a 0.5% greater improvement in HbA1C, but without significant improvements in fasting blood glucose, total cholesterol or blood pressure [55,56].

A recent meta-analysis analyzed the effect of protein intake on the metabolism of T2DM patients. The results showed that there were no significant differences in relation to HDL and LDL cholesterol in the groups with a high-protein and low-protein diet. The same applies to HbA1C. However, significant reductions in blood pressure and greater weight reduction were observed [56].

Researchers who develop high-protein diets can provide positive regulation of anorectic hormones {cholecystokinin (CCK), peptide YY (PYY) and glucagon-like peptide-1 (GLP-1)}, which suppress brain reactions that are linked to increased appetite. Furthermore, protein consumption also helps to increase gastric emptying time, promoting a greater satietogenic effect. In the long term, reduced appetite may contribute to weight loss, blood pressure, and molecular biomarkers. In any case, a protein intake range of 15% to 20% of TEV is the most prevalent in the literature, and so far appears to be the most fair prescription for the management of T2DM. Additionally, more studies are needed [56].

#### 3.2.3. Fats

The Dietary Guidelines for Americans defines a dietary fat prescription with a 25 to 35% TEV fat range. Dietary patterns that recommend replacing saturated fats with polyunsaturated and monounsaturated fats have demonstrated positive results in reducing blood glucose, triglycerides, LDL cholesterol, and increasing HDL cholesterol. The types or quality of fats in dietary plans can influence results associated with a greater likelihood of developing cardiovascular diseases [57,58,59,60].

The American Heart Association (AHA) recommends a low-fat diet, given that the world population consumes, on average, 36–46% of their TEV in fats. This high intake has been associated in several studies with increased cardiovascular risk. Additionally, only for individuals with hypercholesterolemia and T2DM, the American College of Cardiology (ACC) and the AHA recommend limiting the TEV of saturated fats to 5% to 6%. European and Brazilian guidelines recommend limiting the consumption of saturated fats to <7% of the TEV and total fats to <35% of the TEV to control dyslipidemia and T2DM [16,61,62,63].

High-fat diets, especially those rich in saturated fatty acids, are capable of altering the composition of the gut microbiota, causing a decrease in bacterial diversity and an increase in intestinal permeability. This process raises lipopolysaccharides (LPS) and activation of TLR4 (toll-like receptor 4), generating metabolic endotoxemia and low-grade systemic inflammation. This process add to development of several chronic diseases such as obesity, diabetes, and atherosclerosis [64]. In addition, patients also pay attention to trans fatty acids. Foods containing trans fatty acids (hydrogenated vegetable fat) should be minimized as much as possible from the diet [16,33]. Excess trans and saturated fat intake above the recommendation has been associated with a higher risk of T2DM [16,61,64,65], and can also increase the inflammatory response in the gut microbiota [65].

-Cholesterol

The Dietary Guidelines for Americans concluded that the available evidence does not support the recommendation to limit dietary cholesterol for the general population; the exact recommendations for patients with chronic noncommunicable diseases, such as T2DM, are still less clear. Some researchers argue that the body produces enough cholesterol to perform its physiological and structural functions, so that people do not need to obtain it from food [33]. The studies also indicate that dietary cholesterol intake is correlated with an increase in total cholesterol levels, but this is not linked to a greater likelihood of cardiovascular diseases. More research is needed on the relationship between dietary cholesterol, blood cholesterol, and cardiovascular events in people with T2DM [16,60,66].

-Saturated fat

Guidelines recommend that patients with dyslipidemia and T2DM consume an average of <7% of their TEV of saturated fat [16,33,62,63,67]. The scientific justification for reducing saturated fat in the diet is based on the significant effect of saturated fat intake on increasing LDL-C, a factor that contributes to the development and increased risk of atherosclerosis [68]. The American Heart Association concluded that reducing saturated fat intake and replacing it with unsaturated fats, especially polyunsaturated fats, reduces the incidence of cardiovascular disease [69].

A meta-analysis showed a 17% reduction in the risk of cardiovascular events in studies that reduced saturated fat intake from 9% of the TEV, but no reductions in stroke, cardiovascular mortality, or all-cause mortality were found. Benefits have also been seen when replacing saturated fat with polyunsaturated fat, but not with carbohydrates or proteins [70]. In another study, with patients with T2DM, intake of food sources of monounsaturated and polyunsaturated fats was associated with a lower risk of CVD and death, while intake of saturated fat and trans fats was associated with a higher risk of cardiovascular disease. Replacing saturated fat with monounsaturated or polyunsaturated fat and replacing trans fat with monounsaturated fat have been associated with a reduced risk of developing cardiovascular disease [71,72].

In general, replacing saturated fat with unsaturated fats, especially polyunsaturated fat, significantly reduces total cholesterol and LDL-C, and replacing it with monounsaturated fat from plant sources such as olive oil and nuts reduces the risk of cardiovascular disease [67]. Replacing saturated fat with carbohydrates also lowers total cholesterol and LDL-C, but significantly increases triglycerides and lowers HDL-C [68,73].

Recently, there has been an increase in the consumption and prescription of foods that are sources of saturated fats by doctors and nutritionists, so guidelines have taken a strong stance against the use of coconut oil, lard, bacon, and other foods rich in saturated fatty acids. They have high levels of lauric, myristic, and palmitic acid, so daily consumption should be avoided; they should be replaced by vegetable oils rich in monounsaturated and polyunsaturated fatty acids, such as olive oil, canola oil, and sunflower oil [48].

A systematic review and meta-analysis analyzed the effect of coconut oil consumption on LDL-C, HDL-C and other cardiovascular risk factors compared to other cooking oils. The results showed that coconut oil consumption significantly increased LDL-C +10.47 mg/dL and minimally increased HDL-C +4.00 mg/dL. The authors concluded that even when HDL-C is increased, its daily consumption should be avoided, as it results in higher LDL-C than other vegetable oils [74]. Coconut oil is rich in saturated fatty acids, especially lauric acid (12 carbon atoms), myristic acid (14 carbon atoms), and palmitic acid (16 carbon atoms). These types of fatty acids can contribute to increasing concentrations of total cholesterol and LDL-C [74].

Another meta-analysis also demonstrated that all these saturated fatty acids (lauric, myristic, and palmitic acid were included in items such as coconut oil, hydrogenated vegetable fat, and palm oil) increase LDL cholesterol. Therefore, the authors suggest that coconut oil should be replaced with other unsaturated vegetable oils (olive oil, canola oil, and sunflower oil). The results are extremely relevant for nutritional and dietary guidelines [75].

However, other studies did not show an association between intake of some foods that contain a small portion of saturated fat, such as dairy products and eggs, with an increased risk of diabetes and cardiovascular diseases. Dairy products also have carbohydrates and proteins in their composition, and their saturated fat contents would not be too high compared to other sources of saturated fat. Studies suggest that dairy products can be included in the diet of T2DM, as long as they are quantified in <7% of saturated fats in the TEV. Therefore, there should also be more interest in giving preference to low-fat dairy products to ensure that the daily intake of saturated fats does not exceed 7% of the TEV [76,77,78].

A similar result was found for eggs. Although they contain fat, they are also rich in proteins, water, phytochemicals, carotenoids, and micronutrients. Their consumption was not associated with cardiovascular risk, as long as it is properly quantified within the energy need for saturated fats within the TEV (<7% for T2DM). Unfortunately, there is still no consensus on the amount of egg intake per day or week, as there is great divergence in the literature regarding the intake protocols used [51,62,63,68].

Some research has shown an increase in the risk of T2DM in individuals who consumed 3 to 4 eggs per week, and an increase in those who consumed more than 5 eggs per week. Separation of intake by sex was also studied, illustrating that an intake of 5 eggs per week in men and above 7 eggs per week in women could increase the risk of T2DM. Opposite results were observed in other studies, as higher egg consumption was associated with a lower risk of T2DM. In systematic reviews and meta-analyses that evaluated healthy individuals, there was also no consensus on the association between egg consumption and a higher risk of cardiovascular disease or T2DM. Confounding factors between saturated fat intake and the amount of calories ingested per day (which favor weight gain and the development of metabolic syndrome) can limit study results. In any case, more long-term research is needed [62,63].

-Monounsaturated fats

Monounsaturated fatty acids have been recommended in the literature for their great capacity to reduce inflammatory response and cardiovascular risk. Two clinical trials applied the Mediterranean Diet, which is rich in food sources of monounsaturated fatty acids (extra virgin olive oil and walnut oil), and showed a reduction in the incidence of disease, cardiovascular risk, blood glucose, and body weight in T2DM [79,80].

A systematic review and meta-analysis including 1460 participants compared the effect of eating plans high in monounsaturated fat with eating plans high in carbohydrates. Eating plans high in monounsaturated fat showed significant reductions in fasting glucose, triglycerides, body weight and systolic blood pressure, along with significant increases in HDL-C. The researchers also compared eating plans rich in polyunsaturated fat with monounsaturated fat, and found a significant reduction in fasting plasma glucose in both [57].

Canola oil is another excellent source of monounsaturated fatty acids (omega 9), and unfortunately has a bad reputation due to distorted information published in the media. Moreover, it is an economically accessible source of fat, and rich in bioactive compounds. Its consumption is safe, and is assessed through the extremely rigorous processing quality control standards of regulatory bodies in all countries. A systematic review and meta-analysis published in the Journal of American College Of Nutrition analyzed the lipid profile performance of 1359 participants who ingested canola oil. The results showed that its consumption reduced total cholesterol (−7.24 mg/dL) and LDL-C (−6.4 mg/dL) compared to sunflower oil and coconut oil. There were no effects on HDL -C, triglycerides, Apo B and Apo A. These results are extremely relevant for delaying the progression of heart disease [81].

-Polyunsaturated fats

Recommendations suggest increasing intake of food sources of omega-3 polyunsaturated fatty acids (long-chain) containing a good proportion of EPA and DHA, such as salmon, tuna, mackerel, prego fish, anchovies, sardines, hake, algae, seeds, vegetable oils and others. Omega-3 and are recommended for T2DM because they have an anti-inflammatory and antioxidant effects on cytokines and low-density lipoproteins (LDL), and help to prevent heart disease. For vegetarians or vegans, omega-3 α-linoleic acid (ALA) is found in plant-based foods such as olive oil, canola oil, avocado oil, walnut oil, seeds, and algae. They are substitutes for foods high in saturated fat, and provide benefits for preventing and decreasing the risk of cardiovascular disease [16,33,82,83].

Large epidemiological studies have shown the consumption of polyunsaturated fatty acids is associated with a lower risk of T2DM [84]. Furthermore, in a randomized clinical trial with cardiac patients with T2DM, researchers demonstrated the benefits of the addition of 2 g of omega-3, twice a day, for patients on statin therapy, resulting in lower mortality rates from cardiovascular diseases [85,86].

In any case, many studies do not recommend omega-3 supplementation for the prevention or treatment of cardiovascular events and T2DM. A recent clinical trial found that omega-3 supplementation at a dose of 1 g/day did not reduce cardiovascular risk in people with T2DM [86,87,88]. Another research showed that vitamin D supplementation with 1 g of omega-3 in patients with T2DM also did not result in a lower incidence and fewer predictors of major adverse cardiovascular events [89]. Studies are inconclusive, and did not present solid evidence for the reduction of cardiovascular diseases events or mortality, but this may be useful in people who require a reduction in triglycerides and attenuation of inflammatory response [87,89].

A recent study carried out 4 g/day omega-3 supplementation in 823 participants diagnosed with T2DM. The results showed a significant reduction in cardiovascular events by 25% when compared to placebo. However, this dose is very high, which makes this dietary supplementation more expensive. Therefore, further research is needed [16,86].

-Trans fats

The use of trans fats in industry provides advantages to food factories, such as cost reduction, longer shelf life, high melting point, and wide potential for use. However, the literature shows an association between intake and an increase in systemic inflammatory response and cardiovascular risk. Furthermore, a meta-analysis showed that trans fat intake resulted in an increase in total cholesterol and LDL-C, and a decrease in HDL-C concentration [90]. Trans fats have also been associated with all-cause mortality and cardiovascular disease mortality. Therefore, the intake of trans fatty acids should be avoided and excluded from the diets of T2DM patients [13,16,63,71].

#### 3.2.4. Fibers

The Dietary Guidelines for Americans 2020–2025 recommend that patients with T2DM consume at least 14 g of fiber per 1000 kcal (or 28 g per 2000 kcal), which must come from the consumption of grains, whole grains, vegetables, legumes, fruits and legumes (beans, peas, and lentils) [33]. Regular intake of dietary fiber is associated with reduced all-cause mortality in T2DM, and should therefore be encouraged, as it also provides additional benefits in obtaining micronutrients and phytochemicals [36,37,39]. Some studies have shown a reduction in total cholesterol, LDL cholesterol and HbA1C with an intake of 25 g of fiber per day. However, this excessive intake may cause flatulence, bloating, and diarrhea. Therefore, supplementation should occur in specific cases, and is not recommended in the long term [39,91].

#### 3.2.5. Sodium

Many groups of Health Sciences researchers recognize that the average sodium intake of the population is greater than 3500 mg per day, and must be reduced to prevent and control hypertension [26,33,39,92,93,94]. Reducing sodium to the general recommended intake of 2300 mg/day (5 g of table salt) demonstrates positive effects on blood pressure and for patients with T2DM, especially those with hypertension and heart disease [31,95].

However, high reductions in sodium should be avoided, as some studies have shown an increased risk of mortality associated with very low sodium intake. This can be explained by the increased excretion of sodium in urine in T2DM. Therefore, sodium intake targets below 2300 mg/day should be avoided and considered individually according to dietary preference and palatability, using medical monitoring [96,97,98,99].

#### 3.2.6. Alcohol

Guidelines suggest moderation for adults with T2DM who drink alcoholic beverages. It is recommended that healthcare professionals warn patients about the signs, symptoms and self-care of reactive hypoglycemia after drinking alcohol, especially when using hypoglycemic medications, so monitoring glucose after drinking alcoholic beverages should also be encouraged [9,16,33,63].

Some studies demonstrate that moderate alcohol consumption has a minimal, if any, effect on blood glucose in T2DM [100,101,102,103]. The maximum daily intake recommendation is defined as 15 g for women and 30 g for men. This 15 g a day may be represented by a 12-ounce (355 mL) bottle of beer, a 5-ounce (150 mL) glass of wine, or a 1.5-ounce (45 mL) portion of distilled beverages [9,33]. Excessive alcohol use (more than 3 drinks per day or 21 drinks per week for men, and more than 2 drinks per day or 14 drinks per week for women) may contribute to hyperglycemia [9,104,105].

However, studies have shown glycemic and cardiovascular benefits from moderate alcohol consumption, but this needs to be analyzed carefully, because chronic intake can put people with T2DM at risk of reactive hypoglycemia [103,106,107,108,109]. This effect may be the result of inhibition of gluconeogenesis, reduced perception of hypoglycemia due to the cerebral effects of alcohol, and reduced counterregulatory response to hypoglycemia. This is relevant for patients who use insulin secretagogues and may have a fasting hypoglycemia following alcohol consumption at night [32,33]. To minimize the risk of nocturnal hypoglycemia, it is suggested that patients eat some food when they drink alcohol [32,33,109]. It is essential that people with T2DM receive education about recognizing the symptoms and managing reactive hypoglycemia, as well as engaging in frequent blood glucose monitoring after alcohol consumption [32,33,110].

Other reviews and meta-analyses also suggest a protective effect of moderate alcohol intake on the risk of developing T2DM [101,111,112]. Moderate alcohol intake ranging from 6–48 g/day (0.5–3.4 drinks) has been associated with a 30–56% lower incidence of T2DM [32,33,101,110,111,112]. Some studies have found an intake of 20 to 30 g of alcohol per day from wine or beer to decrease the incidence of T2DM by 20% for wine and 9% for beer. However, the authors do not advise that patients who do not drink alcohol should start consuming it [113]. In any case, alcohol consumption is an individual choice, but additional factors such as history, religion, genetic factors, mental health, and drug interactions, should be considered before alcohol use [33].

#### 3.2.7. Sweeteners

The Dietary Guidelines for Americans suggest replacing sugar-sweetened beverages (non-diet soft drinks/sodas, flavored juice drinks, sports drinks, sweetened tea, coffee drinks, energy drinks, and electrolyte replacement drinks) with water [33]. One study found that replacing sugary drinks with an equal amount of water reduced the risk of T2DM by 7–8% [114]. When low-energy sweeteners are used to reduce overall calorie and carbohydrate intake, people should be advised to avoid compensatory behaviors like additional calorie intake from other food sources [33].

Consumption of sugary drinks by the general population contributes to a significantly increased risk of T2DM, weight gain, heart disease, kidney disease, non-alcoholic liver disease, and tooth decay [115]. A meta-analysis reported that consuming one serving of a sugary drink per day increased the risk of T2DM in adults with prediabetes by 26% [116]. Another study showed that regular soda intake increased the risk of T2DM by 13%, while diet soda consumption increased the risk of T2DM by 8% [117].

The Food and Drug Administration (FDA) and Health Surveillance Agency have reviewed the safety of ingesting various types of sweeteners, approving them for consumption by the general public, including T2DM patients. The term “sweeteners” refers to high-intensity sweeteners, artificial sweeteners, non-nutritive sweeteners, and low-calorie sweeteners. These include saccharin, neotame, acesulfame-K, aspartame, sucralose, advantame, stevia, and others. Replacing table sugar and sweetened beverages with sweeteners can reduce carbohydrate and calorie intake. These dietary changes can beneficially affect blood glucose, weight, and cardiometabolic control [118,119].

Unfortunately, there is not enough evidence to determine whether the use of sweeteners could really contribute to weight loss, reduced cardiometabolic risk, and attenuation of glycemia in the long term [118]. Moreover, it may be said that the use of sweeteners is interesting, as long as individuals do not compensate for calories throughout the day. Studies conclude that sweeteners can be useful in reducing caloric intake, especially carbohydrates, but more research is needed [120,121]. Regarding adverse effects, studies have investigated hypotheses regarding (1) changing the sensation of hunger and satiety; (2) excessive use of dietary products and consequent dysregulation of the intestinal microbiota; and (3) reduced perception of calorie intake [118,122]. Therefore, for people who looking to reduce intake of sugary drinks, drinking water is more encouraged than substituting diet drinks [118].

Sugar alcohols/polyols represent a separate category of sweeteners, and have been approved by the FDA for consumption by the general public and in T2DM. Sugar alcohols have fewer calories per gram than sugars, and they are not as sweet. Therefore, a larger amount is needed to match the sweetness of the sugars, generally raising caloric content to a level similar to sugars [123]. The use of sugar alcohols should be moderate, as they can cause gastrointestinal effects in sensitive individuals. Currently, there is little research on the benefits of sugar alcohols for people with T2DM [9,124].

#### 3.2.8. Micronutrients and Supplements

The benefits of multivitamins or mineral supplements on glycemia and cardiovascular disease risk in T2DM have little literary support, and routine use is not recommended for people who are not deficient [22,125,126]. However, metformin is associated with vitamin B12 (B12) deficiency, and annual monitoring of blood levels are recommended for people who are undergoing therapy, especially those who are anemic or have peripheral neuropathy [127], because metformin can reduce absorption of B12 [128,129,130,131].

The use of micronutrient and nutraceutical supplements, such as chromium, chromium picolinate, L-carnitine, zinc, propolis, spirulina, chorella, vitamin D, cinnamon, curcumin, aloe vera, coconut oil, cardamom oil or any other supplements to improve blood glucose in T2DM is not supported by evidence and is not recommended [9,16,48,125,126,131]. Patients who do not meet glucose targets may be at increased risk of micronutrient deficiencies [126]. Therefore, it is essential to maintain a balanced intake of food sources that provide at least the Recommended Dietary Allowance [22]. For pregnant, celiac, elderly, and vegetarian patients and those who wish to lose weight, supplementation may be fair and adjusted for each clinical case [126,132,133].

A systematic review on the effect of chromium supplementation on glucose and lipid metabolism concluded that the evidence is limited by poor-quality primary studies and heterogeneity in methodology [133,134]. Evidence from clinical studies evaluating magnesium and vitamin D supplementation to improve blood glucose in T2DM is also equally contradictory [134,135,136,137,138]. Clinical trials and meta-analyses that evaluated vitamin D supplementation to improve glycemia in T2DM have concluded that prescription is unnecessary in patients who are not deficient. Those who are deficient must be supplemented and monitored every 6 months [137,138,139,140,141,142,143,144,145,146].

Creatine has been gaining prominence in the literature within the treatment of T2DM. Evidence suggests that creatine supplementation alone or in combination with exercise training can reduce glucose intolerance and insulin resistance in T2DM. The mechanism of the effects is an increase in glucose transport into the muscle cells through translocation of glucose transporter type 4 (GLUT-4) to the sarcolemma. It is believed that creatine increases the amount and speed of translocation too. The evidence is considered promising, but more research is needed [147].

In any case, some nutritional supplements, herbal, and nutraceutical products are not yet regulated by supervisors and standard-setting bodies. Health professionals should consider the real costs, benefits, adverse effects, and drug interactions. The variability of herbal and micronutrient supplements makes research challenging, making it difficult to conclude on their effectiveness. Unfortunately, there is limited evidence supporting the use of supplements and nutraceuticals to control blood glucose [148,149].

### 3.3. Nutritional Strategies

Many types of dietary patterns for the nutritional management of T2DM have been studied, but the scientific community always highlights: (1) increased intake of fruits, vegetables, and legumes; (2) minimizing added sugars and refined grains; (3) choosing natural and minimally processed foods rather than ultra-processed foods [13,22,132].

#### 3.3.1. Mediterranean Diet

The Mediterranean diet is an intervention that has the highest level of scientific evidence, and consists of foods of high nutritional quality, such as fruits, vegetables, legumes, seeds, whole grains, fish, lean meats, skimmed dairy products, and olive oil. Several epidemiological studies have reported the protective effect of this diet on metabolic disorders, chronic diseases, and mental health. This diet has good adherence in the long term, and is recommended within the prescription of individualized, flexible, and balanced eating plans that match the patient’s goals [9,16,132,150].

Studies show that the Mediterranean lifestyle with a low-fat or low-carb diet offers multiple benefits to T2DM patients [79,150,151,152,153]. A randomized controlled trial compared the Mediterranean diet (low-carb) to the low-fat diet, aiming to analyze the prevention of T2DM. The results showed that the Mediterranean diet resulted in a 30% lower relative risk compared to the low-fat diet [80]. Moreover, epidemiological studies correlate the Mediterranean, vegetarian and DASH (Diet Approach to Stop Hypertension) diets with a lower risk of developing T2DM, with no effect shown for the Ketogenic diet in the long term [115,154,155,156,157,158,159,160,161,162,163,164,165]. Large clinical trials show that a low-fat diet optimizes weight loss and improves glucose tolerance, and causes a decrease in the incidence of T2DM [151,152,160,161].

Studies have also pointed out that the Mediterranean diet may have a mixed effect on HbA1C, weight and cholesterol. In a clinical trial, obese patients with T2DM were divided in to three groups: the Mediterranean diet with calorie restriction vs. a low-fat diet with calorie restriction vs. a very low-carbohydrate diet (28% of TEV carbohydrates) with calorie restriction. The results showed that fasting glucose was lower in the Mediterranean diet than in the low-fat and very low-carb groups [165].

Another study compared the Mediterranean diet with the low-fat diet for 4 years. The results showed an improvement in the control of the glycemic profile in the Mediterranean diet, and the need for the use of hypoglycemic medications was lower too [166]. Another study showed that a Mediterranean diet with olive oil and nuts significantly reduced the incidence of cardiovascular disease in T2DM patients [29].

A randomized crossover study evaluated the impact of the Ketogenic diet vs. the Mediterranean diet on T2DM. Both diets incorporate the inclusion of non-starchy vegetables, the restriction of added sugars, and limitation of refined grains. The main differences are the consumption of legumes, fruits, and whole grains only in the Mediterranean. The authors concluded that both diets produced beneficial effects for individuals and a significant reduction in HbA1c, but the ketogenic diet increased LDL, making it impossible to rule out the potential cardiovascular risks that this change presents; in addition, it reduced intake of vitamins and minerals, which is closely correlated with the restriction of important food groups. Although controlling and reducing carbohydrate intake is beneficial and recommended for controlling prediabetes and T2DM, more studies are needed [167].

#### 3.3.2. Dash (Dietary Approaches to Stop Hypertension)

The DASH diet is an intervention whose objective is weight loss and consequently a reduction in blood pressure. It has characteristics similar to the Mediterranean pattern, but alcohol consumption is not encouraged, and there is also sodium restriction. One study found that the DASH diet can reduce HbA1C, blood pressure, total cholesterol and weight levels in T2DM, but without significant differences in triglycerides [132,168].

Another study compared the DASH diet with conventional restriction. The results showed that blood pressure and weight were lower in the DASH group, but HbA1C and lipids showed no statistically significant differences [30,31,95]. In any case, guidelines have stated that DASH and the Mediterranean diet can be effective in the management of T2DM, especially for those who also have hypertension and coronary heart disease [16,63].

#### 3.3.3. Low-Carb Diet and Ketogenic Diet (Very Low-Carb Diet)

Low-carb diets, especially the very low-carb (Ketogenic) diet were initially proposed for the management of patients with epilepsy and autism. Over the years, there has been interest from the scientific community in expanding these diets to treat other diseases, especially T2DM. Despite several different types of protocol that define the low-carb diet, the standard that most prevails in the literature has wide variability in the consumption of fresh and minimally processed foods, with high nutritional quality. Dietary prescription is also variable, but in general is <40% TEV carbohydrates, 20–25% TEV proteins, 30–40% TEV fats, and a limit of <10% saturated fats in most studies [9,16,132].

Part of the challenge in interpreting low-carb research is the wide range of definitions of a low-carb diet. Weight reduction is also an objective outlined in many studies, which further complicates the assessment of the distinct contribution of dietary patterns, as weight loss can also have positive effects on patients with T2DM [9]. Clinical trials have shown that this diet could reduce HbA1C and the need for antihyperglycemic medications. A meta-analysis compared the low-carb (≤45% of carbohydrate TEV) and high-carbohydrate diets (>45% of carbohydrate TEV). Both groups restricted saturated fat intake to <10% of TEV. The results showed that the benefits of improving HbA1C were more pronounced in individuals who followed the low-carb diet [168,169].

Other interventions also showed benefits in improving HbA1C in patients with T2DM who followed a Ketogenic diet (<26% of TEV in carbohydrates) for 3 and 6 months. However, the same results were not found at 12 and 24 months. The authors discuss that the major limitations of these strategies would be low adherence to the diet and the increased likelihood of long-term loss of lean mass. Food sources of carbohydrates are hyperpalatable, as are fats, and alongside providing energy substrates for physiological and metabolic functions, they also have an important emotional role. The production of ketone bodies would not be sufficient to sustain the myocyte’s energy demand during long-term muscle contraction. Therefore, researchers suggest individualized adjustments and flexibility after 3–6 months of following this type of intervention, for greater adherence to lifestyle changes [9,170].

Another meta-analysis compared the low-carb (<40% of the TEV from carbohydrates) and low-fat diets (<30% TEV from fat) for 6 months. All groups restricted saturated fat intake to <10% of the TEV. The results showed that the low-carb diet reduced HbA1C, triglycerides, blood pressure, and the use of hypoglycemic drugs, and increased HDL-C [171]. Another study compared the low-carb diet and high-carbohydrate diet (both groups restricted saturated fat intake to <10% of TEV), and the results showed a reduction in HbA1C, but this was not sustained in the long term [172].

However, the ketogenic diet is not recommended for people with T2DM who have chronic kidney disease, people with eating disorders, or women who are pregnant. More studies and literary support are needed before recommending this diet. Adopting the very low-carb diet can cause diuresis and quickly reduce blood glucose levels. Therefore, multidisciplinary team management is necessary to prevent dehydration and hypoglycemia [9,16,132].

Reducing carbohydrate intake in T2DM appears improve glucose metabolism and molecular markers, thereby becoming a viable nutritional strategy. However, studies on ketogenic diets generally indicate challenges to long-term sustainability. Therefore, it is important to reevaluate and individualize eating plan guidance regularly for those interested in this approach. It is worth remembering that insulin and other medications may need to be adjusted to prevent hypoglycemia and blood pressure [9,132].

In any case, no randomized trials have been performed with people with T2DM who increased saturated fat intake on low-carb or ketogenic diets to examine the effects on blood glucose and risk factors for cardiovascular disease [167,171,172,173,174]. Guidelines recommend the restriction of saturated fats, comprising 5–7% of the TEV for patients with T2DM [9,16,47,48,49,68]. Therefore, more evidence is needed to analyze the real efficacy, adherence, improvement of biochemical and cardiovascular parameters in the long term [167,170,171,172,173,174].

#### 3.3.4. Low-Fat Diet

The Look AHEAD (Action for Health in Diabetes) research group showed in studies that patients who followed low-fat calorie restrictions achieved positive scores compared to a conventional calorie-restricted diet [175,176]. The evidence has shown the several benefits of a low-fat diet in improving biochemical markers (glucose, glycated hemoglobin, and LDL cholesterol), cardiovascular markers, and even quality of life [3,6,7,8,11,16]. However, according to some studies, reducing total fat intake did not improve blood glucose and cardiovascular disease risk factors in people with T2DM compared to conventional calorie restriction [177,178,179,180,181]. The benefit of the low-fat diet appears to be primarily related to weight loss [16,175,176].

The American Heart Association, the Brazilian Society of Cardiology, and the European Guidelines emphasize that low-fat diets, especially a Mediterranean or vegetarian dietary patterns, are very important for adapting the consumption of fats and fiber by the population; they are potentially efficient strategies, especially for patients who have increased LDL cholesterol. Currently, individuals consume a large amount of fat (>36–46% of TEV), and this excess has important consequences, in particular increased cardiovascular risk and weight gain. Therefore, this strategy is also viable for the management of T2DM [16,60,61,62,63,64,74,75,132].

#### 3.3.5. Ornish and Pritikin Diets (Very Low-Fat Diet)

Both of these types of diets are very low-fat eating patterns. The Ornish program emphasizes a low-fat, whole-food, plant-based eating plan (70% TEV carbohydrates, 10% TEV fat, 20% TEV protein, and 60 g fiber per day) predominantly made up of vegetables, legumes, fruits, grains, skimmed dairy, and egg whites [182,183,184]. In the same way, the Pritikin diet recommends the consumption of 77% carbohydrates, 10% fat, 13% protein within the TEV, and 30–40 g of fiber without caloric restriction during a 26-day stay in a treatment center (SPA). A meta-analysis with 652 participants showed that these diets can have positive effects on glucose levels, weight, blood pressure, and HDL-C in the long term [182,183,184,185]. Unfortunately, these studies are old, and interest in studying these types of interventions has reduced over time, so more evidence is needed [16,132].

#### 3.3.6. Vegetarian and Vegan Diet (Plant-Based Diet)

Studies of vegetarian or vegan eating plans over 12 to 74 weeks have demonstrated positive results on blood glucose, risk of cardiovascular disease, and weight loss. Two meta-analyses concluded that vegetarian and vegan diets can reduce HbA1C, weight, waist circumference and LDL-C in patients with T2DM. No significant effects were found on HDL-C, insulin, triglycerides, and blood pressure [16,132,184,185,186,187,188,189,190,191].

Studies have demonstrated the benefits of plant-based diets in treating T2DM and reducing macro and microvascular complications. The type and source of carbohydrates (unrefined vs. refined), fats (monounsaturated and polyunsaturated vs. saturated and trans), and proteins (plant vs. animal) play an important role in preventing and controlling disease, and have multiple benefits such as improving insulin resistance, reducing body weight, increasing fiber and phytonutrients, increasing interactions between food and the microbiome, decreasing saturated fat, and advancing glycation end-products and nitrosamines [16,132,184,185,186,187,188,189,190,191].

#### 3.3.7. Paleolithic Diet (Paleo Diet)

The Paleolithic diet is an intervention made to mimic the eating habits of our ancestors. Unfortunately, Westernization makes it difficult to follow this strategy, and it is not applicable in the 21st century. It is worth remembering the individuals who lived in the Paleozoic era were hunters, gatherers, and nomadic peoples. They consumed raw meat, fruits, vegetables, and roots, and they survived from what nature could offer and spent long periods fasting [192,193,194].

This lifestyle has become impossible, especially due to eating raw meat and high levels of food contamination. Studies using the Paleolithic diet in T2DM are scarce. They have a short duration (3 months), few participants (*n* = 13–29), and other methodological limitations. The most studies findings found mixed effects on HbA1C, weight, and lipids [193,194]. Due to the lack of literature, this dietary pattern is not recommended by guidelines [16,47,132].

#### 3.3.8. Intermittent Fasting

Intermittent fasting is not an eating pattern by definition, but it was included in this literature review due to increased interest from the scientific community. Fasting means going without eating and drinking and abstaining from foods and drinks that contain macronutrients and calories. People fast for any reasons, such as rapid weight loss, and religious or spiritual practices. Intermittent fasting is a strategy that focuses more on time, i.e., “when” you eat, rather than “what” you eat. Daily caloric intake occurs within a defined dietary window during the day [195].

Studies on intermittent fasting with T2DM demonstrate a variety of protocols: (1) restriction of food intake for 18–20 h a day; (2) fasting all day (24 h fasting with days of normocaloric intake); and (3) severe calorie restriction (intake < 1000 kcal/day) for up to 8 consecutive days or more [196]. Some clinical trials with few participants (≤63 participants) and a short duration (≤20 weeks) have demonstrated that fasting protocols on consecutive days with severe caloric restriction (<1000 kcal/day) and fasting > 16 h per day can result in weight loss in T2DM. However, there were no improvements in HbA1C compared with conventional calorie restriction. One of the studies showed modest reductions in HbA1C, weight, and medication doses when patients underwent 2 days of severe energy restriction compared to conventional energy restriction [195,196,197].

A study has analyzed the applicability of fasting in patients with pre-diabetes. The individuals were divided into two groups: (1) intervention with a dietary window of just 6 h (last meal at 3 p.m.); and (2) a control group with a 12 h food window. The results showed improved insulin sensitivity, greater responsiveness of β cells, and a reduction in blood pressure and oxidative stress in the intervention group. However, the study did not control diet and exercise [197].

The results of intermittent fasting in T2DM are still controversial, and have limitations (short-term studies, few participants, low long-term adherence, lack of control over diet and physical exercise). Therefore, its practice requires great caution, especially for patients who use hypoglycemic medications [197]. The safety of fasting in other specific subgroups, including pregnant women and patients with eating disorders, has not yet been studied and is also not recommended [16,47,132].

#### 3.3.9. Mindful Eating Program

The ‘Mindful Eating Program’ has also shown strong evidence for the treatment of different diseases, even T2DM. This therapy mixes the science of nutrition, body awareness, and self-control. Studies have demonstrated significant improvement in eating regulation in T2DM, as prioritizing self-control can be a great alternative to severely restrictive diets. The practices can also help to increase knowledge of the factors (physiological, environmental, or emotional) that dictate excess food intake and train patients in intuitive eating (respecting hunger/satiety signals and chewing food). The program not only emphasizes the quantity and quality eaten, but also the enjoyment of the experience, thus changing the reward value of the food without restricting it [198,199].

The Mindful Eating intervention has been gaining increasing prominence in the scientific community, as it is closely linked to mental health care [198,199]. The American Diabetes Association emphasizes that the psychological factors of patients with T2DM must be respected and valued by health professionals. Therefore, it is extremely important to welcome and understand what happens on an emotional, physiological and metabolic level. More evidence is needed on applicability in T2DM in the long term [16,132,198,199].

### 3.4. Summary of Dietary Patterns for the Management of T2DM

Table 1 below summarizes all descriptions of dietary patterns, their level of evidence, adherence, and comments.

### 3.5. Nutritional Management of T2DM in the COVID-19 Pandemic

The COVID-19 lockdown clearly affected the lifestyle of the population and entailed changes in their daily habits. These transformations involved potential health consequences, especially in T2DM. Recent studies have examined the impact of lockdown on eating habits, exercise, and the psychological effects in T2DM, and showed that there was an increase in the consumption of foods high in sugar and sandwiches during the pandemic. An association between food craving levels and sandwich consumption was also found. The research also showed a high prevalence of physical inactivity before lockdown, which was even higher during home confinement. These findings emphasize the great importance of developing new research with larger samples, aiming to expand public health policies that promote a healthy lifestyle in T2DM, especially after lockdown [200].

However, another study shows the more than 60% of participants with T2DM and T1DM started eating more nutritious meals at the pandemic, and increased their personal and environmental hygiene habits, especially through the use of hand sanitizers. Additionally, 40% of all respondents stated that their glycemic self-management had improved. These positive results may be related to patients’ fear of knowing that they belong to a risk group, thus having a high probability of suffering with COVID-19. However, the data collection has limitations, because they were obtained through patient reports [201].

Another study found no changes in physical activity and adherence to diet in more than 80% of participants. There was an increase in the consumption of vegetables (81%) and fruits (43%), and a decrease unhealthy snacks (63%). No significant changes were observed in HbA1c or body weight before and after lockdown. The majority watched television and spent time with their families. However, the quality of food intake and glycemic control worsened in those with mental stress, insufficient sleep and physical inactivity [202]. Therefore, it is concluded that measures to promote healthy lifestyle practices, along with ways to reduce psychosocial stress, should be implemented to better management T2DM after the pandemic [200,202].

## 4. Authors’ Comments—The Best Nutritional Strategies and Dietary Prescription Recommended for the Management of T2DM

Based on the data analyzed, the Mediterranean Diet is the nutritional strategy with the highest level of evidence in the literature, and can bring greater benefits to patients with T2DM in the long term. The best recommended dietary prescription is composed of 40–50% carbohydrates, 15–25% proteins, 25–35% fats (<7% saturated, 10% polyunsaturated, and 10% monounsaturated), at least 14 g of fiber for each 1000 kcal consumed, and <2300 mg of sodium. In any case, it is necessary to individualize the diet based on the stage of the disease, the patient’s goals, socioeconomic situation, and preferences, aiming for greater adherence and significant changes in lifestyle.

## 5. Conclusions

There is no consensus on what the ideal nutritional strategy and percentages of calories, carbohydrates, proteins, and fats for patients with T2DM. Therefore, the type of strategy and distribution of macronutrients should be based on an individualized assessment of current eating patterns, preferences, and metabolic goals. However, several references show that Mediterranean diet may bring greater benefits in the long term, with the following recommended dietary prescription: 40–50% carbohydrates; 15–25% proteins; 25–35% fats (<7% saturated, 10% polyunsaturated, and 10% monounsaturated); at least 14 g of fiber for every 1000 kcal consumed; and <2300 mg sodium.

## Figures and Tables

**Table 1:** Summary of dietary patterns for the management of T2DM.

Dietary Patterns	Description	Level of Evidence	Adherence and Comments
Mediterranean Diet	Mediterranean diet encourages consumption of foods with high nutritional quality, such as fruits, vegetables, legumes, whole grains, cereals, tubers, roots fish, low-fat dairy products, vegetable oils, nuts, seed oils, and wine in moderation [16,132,163,164,165,166,167].	High	(1) High long-term adherence [16,132,163,164,165,166,167]. (2) There is a lot of evidence in the literature for the improvement of molecular markers, glycemic control, cardiovascular health, and weight loss [16,132,163,164,165,166,167].
Dash Diet (Dietary Approaches to Stop Hypertension)	DASH diet is similar to the Mediterranean pattern, but alcohol consumption is not encouraged and there is also sodium restriction. It consists of fruits, vegetables, legumes, whole grains, cereals, tubers, roots, fish, chicken breast, lean meats, low-fat dairy products, vegetable oils, nuts, oilseeds and sodium restriction (<2300 mg or 1500 mg for severe heart disease) [16,30,31,63,95,132].	High	(1) High long-term adherence [16,30,31,63,95,132]. (2) There is a lot of evidence in the literature for the improvement of molecular markers, glycemic control, cardiovascular health, reduce blood pressure and weight loss [16,30,31,63,95,132]. (3) Severe sodium restriction (1500 mg) requires caution and monitoring, and is indicated for some specific cases [16,30,31,63,94,95,96,97,98,99,132].
Ketogenic Diet (very low-carb diet)	A dietary pattern composed of 5–10% carbohydrates, 15–25% proteins and 60–70% fats of the total energy value (TEV). Includes raw vegetables, very low-carb fruits (avocado and strawberries), all types of meat (beef, pork, fish and chicken), full-fat dairy products, vegetable oils, animal fat and eggs. It has a low intake of fruits, legumes, whole grains, cereals, roots and tubers [16,132,167,173].	Low/ Moderate	(1) Low long-term adherence [16,132,167,168,169,170,171,172,173,174]. (2) There are many controversies in the literature [16,132,167,168,169,170,171,172,173,174]. (3) No advantages over other nutritional strategies [16,132,167]. (4) The severe carbohydrate restriction may not really be necessary and safe [16,132,167]. (5) New evidence shows results that it improves weight loss and glycemic control in the short term, but increases markers of cardiovascular risk, such as LDL cholesterol [16,132,167]. (6) This dietary pattern also can increase the risk of dehydration and hypoglycemia; there is a high probability of food monotony, low fiber and micronutrients intake [16,132,167]. (7) More studies are needed in patients with diabetes, especially in the long term [16,132,167].
Low-Carb Diet	Low-carb diet promotes reducing the consumption of ultra-processed foods. The carbohydrate intake range is 40–45% of the TEV. Encourages the consumption of fruits, vegetables, legumes, whole grains, tubers, fish, lean meats, skimmed dairy products, vegetable oils, nuts, avocados, eggs and seed oils. Carbohydrates of high nutritional quality are allowed, but without excess [16,132,167,168,169,170,171,172,173,174].	High	(1) High long-term adherence [16,132,167]. (2) In recent years, good evidence has been published in the literature for the modulation of molecular markers, glycemic control, cardiovascular health and weight loss [16,132,167,168,169,170,171,172,173,174]. (3) There are several types of protocols, and those that restrict saturated fats (<7% of TEV) and prioritize sources of polyunsaturated and monounsaturated fats show good results [16,132,167,168,169,170,171,172,173,174].
Low-Fat	This dietary pattern involves ingesting 25–30% fats within the TEV. It encourages the consumption of fruits, vegetables, legumes, whole grains, tubers, fish, chicken breast, lean meats, and skimmed dairy products. This eating pattern is similar to DASH [175,176,177,178,179,180,181].	High	(1) High long-term adherence [175,176,177,178,179,180,181]. (2) There is a lot of evidence in the literature for the modulation of molecular markers, glycemic control, cardiovascular health, reduced blood pressure, and weight loss [175,176,177,178,179,180,181].
Ornish and Pritikins (very low-fat diet)	Both eating patterns have very low fat consumption (10% fat of the TEV). They encourage the consumption of whole foods, vegetables, legumes, fruits, grains, low-fat dairy products, and egg whites [16,132,182,183,184,185].	Low	(1) Low long-term adherence [16,132,182,183,184,185]. (2) Over the years, the scientific community has lost interest in studying this type of diet, since it has low adherence, small palatability, and can possibly cause metabolic damage (hormone production, protection, and energy storage) [16,132,182,183,184,185].
Plant-Based Diet (or vegetarian/ vegan diet)	Plant-based diet consisting eat of foods with high nutritional quality, such as fruits, vegetables, legumes, whole grains, cereal, tubers, roots, vegetable oils, nuts and seed oils. In this diet there is no intake of any type of food from an animal source (intake of high-fiber foods). It encourages questioning about food choices, autonomy, and ethical and cultural issues [16,132,184,185,186,187,188,189,190,191].	High	(1) Moderate long-term adherence [16,132,184,185,186,187,188,189,190,191]. (2) There is a lot of evidence in the literature for the modulation of molecular markers, glycemic control, cardiovascular health, reduced blood pressure, and weight loss (intake of high-fiber foods) [16,132,184,185,186,187,188,189,190,191]. (3) However, this diet needs constant nutritional monitoring, because in the long term it can reduce intake of some micronutrients, such as iron, calcium, and vitamin B12 [16,132,184,185,186,187,188,189,190,191].
Paleolithic Diet	The Paleolithic diet consists of following similar eating habits as our ancestors. It encourages the consumption of all types of meat, animal fat, fruits, vegetables, roots, raw foods and all types of food that nature can offer. The habit of fasting is also recommended in this type of dietary pattern [16,132,192,193,194].	Low	(1) Low long-term adherence [16,132,192,193,194]. (2) It has become technically impossible to follow a diet identical to the Paleolithic period in Westernized society [16,132,192,193,194]. (3) More studies are needed in patients with Diabetes [16,132,192,193,194].
Intermittent Fasting	Fasting means abstaining from foods and drinks that contain macronutrients and calories. Daily caloric intake occurs within a defined eating window during the day, and there are several types of fasting protocols (16 to 24 h), aiming to enhance the production of ketone bodies [16,132,195,196,197].	Low	(1) Low long-term adherence [16,132,195,196,197]. (2) It can increase the risk of dehydration, headache, hypoglycemia, and lack of glycemic control [16,132,195,196,197]. (3) It is strongly discouraged by guidelines for patients with diabetes, mainly in diabetic ketoacidosis [16,132,195,196,197]. (4) More studies are needed in patients with diabetes [16,132,195,196,197].
Mindful Eating	The practices of Mindful Eating can help increase knowledge of the factors (physiological, environmental, or emotional) that lead to excessive food consumption and training in intuitive eating (respecting signs of hunger/satiety and chewing food). The program not only values the quantity and quality consumed, but also the pleasure of the experience, thereby changing the value of the food reward without restricting it [16,132,198,199].	Moderate	(1) Moderate long-term adherence [16,132,198,199]. (2) It is a type of nutritional strategy that has been gaining prominence in the literature with good results [16,132,198,199]. (3) However, more studies are needed to evaluate the impact of mindful eating on weight loss, glycemic control, and improvement in cardiovascular markers [16,132,198,199]. (4) Seems to be a good strategy to take care of the subjective issues of nutritional management [16,132,198,199].

## Data Availability

Not applicable.
